# Identification of Genes Responsive to Solar Simulated UV Radiation in Human Monocyte-Derived Dendritic Cells

**DOI:** 10.1371/journal.pone.0006735

**Published:** 2009-08-26

**Authors:** Hortensia de la Fuente, Amalia Lamana, María Mittelbrunn, Silvia Perez-Gala, Salvador Gonzalez, Amaro García-Diez, Miguel Vega, Francisco Sanchez-Madrid

**Affiliations:** 1 Servicio de Inmunología, Hospital de la Princesa, Universidad Autónoma de Madrid, Madrid, Spain; 2 Fundación Centro Nacional de Investigaciones Cardiovasculares Carlos III (CNIC), Madrid, Spain; 3 Servicio de Dermatología, Hospital de la Princesa, Universidad Autónoma de Madrid, Madrid, Spain; 4 Dermatology Service, Memorial Sloan-Kettering Cancer Center, New York, New York, United States of America; 5 Centro de Investigaciones Biológicas (CIB-CSIC), Madrid, Spain; National Institute on Aging, United States of America

## Abstract

Ultraviolet (UV) irradiation has profound effects on the skin and the systemic immune system. Several effects of UV radiation on Dendritic cells (DCs) functions have been described. However, gene expression changes induced by UV radiation in DCs have not been addressed before. In this report, we irradiated human monocyte-derived DCs with solar-simulated UVA/UVB and analyzed regulated genes on human whole genome arrays. Results were validated by RT-PCR and further analyzed by Gene Set Enrichment Analysis (GSEA). Solar-simulated UV radiation up-regulated expression of genes involved in cellular stress and inflammation, and down-regulated genes involved in chemotaxis, vesicular transport and RNA processing. Twenty four genes were selected for comparison by RT-PCR with similarly treated human primary keratinocytes and human melanocytes. Several genes involved in the regulation of the immune response were differentially regulated in UVA/UVB irradiated human monocyte-derived DCs, such as protein tyrosine phosphatase, receptor type E (PTPRE), thrombospondin-1 (THBS1), inducible costimulator ligand (ICOSL), galectins, Src-like adapter protein (SLA), IL-10 and CCR7. These results indicate that UV-exposure triggers the regulation of a complex gene repertoire involved in human-DC–mediated immune responses.

## Introduction

Dendritic cells (DCs) are highly specialized antigen-presenting cells that sit at the crossroads of innate and adaptive immunity and play essential roles in immunity and tolerance. DCs are present in peripheral tissues, where they act as sentries, capturing antigens for presentation to CD4+ and CD8+ T cells. Maturation of DCs is induced upon sensing pathogens, exposure to proinflammatory cytokines or ligation of CD40. Activated DCs alter the pattern of migration receptors (i.e. through up-regulation of CCR7 expression), up-regulate costimulatory and major histocompatibility complex molecules, and secrete cytokines and chemokines that initiate or enhance many T lymphocyte responses [1]. DCs play key roles in the development of antigen-specific effector cells of the T-helper type 1 and 2 lineages and the recently identified Th17 lineage [2], [3], as well as in the induction of regulatory T cells.

Under normal conditions, most peripheral DCs have an immature phenotype; they express low levels of MHC class II and costimulatory molecules and thus cannot productively activate naïve T cells. Beside their roles in antigen presentation and costimulation of naïve T cells, DCs are also important mediators of peripheral immune tolerance and contribute to the maintenance of immune homeostasis [4]. Historically, immature DCs were thought to be mostly non-inflammatory or tolerogenic, whereas mature DCs were considered capable of eliciting proinflammatory responses. Though generally correct, this view now appears to be an oversimplification [5]. DC tolerogenicity seems to be neither the specific property of one DC subset nor to be restricted to immature DCs. Moreover, DC tolerogenicity has been shown to involve several processes, including resistance to maturation-inducing factors, production of soluble factors such as IL-10, and activation of enzymes such as indoleamine 2,3-dioxygenase [4]. In spite of the importance of DCs as APC, our knowledge of molecules expressed in DC that might be involved in the final outcome of the immune response (tolerance or inflammation) is far from complete.

One of the most potent suppressors of immune responses is UV irradiation. Exposure to UV radiation leads to erythema and edema, as well as the initiation of skin neoplasms that would normally be immunologically eliminated [6]. These neoplasms are likely to develop because exposure of skin to UVB radiation also dampens the immune responses that would destroy them. UVB exposure has been shown to suppress immune responses to a variety of antigens, including microorganisms. Another example of UV-induced immunomodulation is the use of phototherapy to treat T-cell mediated dermatoses [7]. The impact on the immune response of UVA, which represents about 95% of environmental UV radiation, has been less studied, though recent studies have highlighted the role of UVA in UV-induced immune suppression [7]–[10].

UV radiation penetrates to the upper dermis, causing cellular and molecular lesions in DCs located here (Langerhans cells and dermal DCs). The effect of UV radiation on DCs has been studied both *in vivo* and in *vitro* [11]–[13]. Recently, several groups have reported the effect of solar-simulated UV radiation on the phenotype and function of human monocyte-derived DCs. Solar-simulated UV radiation results in defective DC maturation and an anomalous migratory phenotype [11], [14]. However, gene expression changes induced in DCs in response to UVA/UVB exposure have not been systematically examined. The aim of this study was to identify genes whose expression is specifically up-regulated or down-regulated in DCs in response to solar simulated-UV radiation, paying special attention to genes potentially involved in the regulation of the immune response.

## Results

### Genes regulated by solar simulated-UV radiation in human monocyte-derived DCs

To identify genes in human monocyte-derived DCs whose expression is up- or down-regulated after exposure to UVA/UVB, we screened RNA probes on a human whole genome microarray. Human monocyte-derived DCs were purified from buffy coats obtained from 3 healthy donors and irradiated with 3.7 J/cm^2^ UVA+0.3 J/cm^2^ UVB, using a solar simulator. This irradiation dose induces the secretion of TNF-alpha and modifies the expression of several DC surface receptors without causing significant hypodiploidy [14]. Statistical analyses of gene expression values of non-irradiated versus irradiated DC, revealed the modulation of 64 annotated genes with p<0.05. Among these 64 genes, 40 were induced>2.0-fold by UVA/UVB, these genes are enumerated in Table 1. Similarly, there were 24 genes that were suppressed>2.0-fold by UVA/UVB listed in Table 2.

**Table 1:** Upregulated genes in UV irradiated Dcs vs non irradiated DCs.

Gene symbol	Access GenID	Name	a Fold change (log 2)
GDF15	9518	Growth differentiation factor 15	5.99
IL1B	3553	Interleukin 1, beta (IL1B)	3.98
FDXR	2232	Ferrodoxin reductase	4.06
PLK2	10769	Polo-like kinase 2	4.32
IL1A	3552	Interleukin 1, alpha	3.59
CXCL2	2920	Chemokine (C-X-C motif) ligand 2	3.52
ULK4	54986	Unc-51-like kinase 4	4.14
IFI27	3429	Interferon, alpha-inducible protein 27	3.23
IL8	3576	Interleukin 8	3.18
CDGAP	57514	Cdc42 GTPase-activating protein	3.1
JARID2	3720	Jumonji, AT rich interactive domain 2	3.36
THBS1	7057	Thrombospondin 1	3.26
DIRAS3	9077	DIRAS family, GTP-binding RAS-like 3	3.17
DOT1L	84444	DOT1-like, histone H3 methyltransferase	3.17
PHLDA2	7262	Pleckstrin homology-like domain, family A, member 2	2.71
PCNA	5111	Proliferating cell nuclear antigen	3.16
CD163	9332	CD163 molecule	2.71
FAM135A	57579	Family with sequence similarity 135, member A	2.93
CCL7	6354	Chemokine (C-C motif) ligand 7	2.5
TMEM88	92162	Transmembrane protein 88	2.42
PARP16	54956	Poly (ADP-ribose) polymerase family member 16	3.84
MYO5B	4645	Myosin VB	2.58
CDC42EP3	10602	CDC42 effector protein (Rho GTPase binding) 3	2.60
NOL7	51406	Nucleolar protein 7, 27kDa	2.38
PCDH12	51294	Protocadherin 12	2.71
HIST1H2BD	3017	Histone cluster 2, H2bd	2.60
RRAD	6236	Ras-related associated	2.49
AICDA	57379	Activation-induced cytidine deaminase	2.25
ETS2	2114	V-ets erythroblastosis virus E26 oncogene homolog 2	2.28
JHDM1D	80853	Jumonji C domain containing histone demethylase 1 homolog D	2.27
HIST2H2BE	8349	Histone cluster 2, H2be	2.27
RCP9	27297	Calcitonin gene-related peptide-receptor component protein	2.66
PAQR6	79957	Progestin and adipoQ receptor family member VI	2.17
CCNL1	57018	Cyclin L1	2.09
RAPGEF6	51735	Rap guanine nucleotide exchange factor (GEF) 6	2.13
PMAIP1	5366	Phorbol-12myristate-13-acetate-induced protein 1	2.10
PICALM	8301	Phosphatidylinositol binding clathrin assembly protein	2.44
RBM16	22828	RNA binding motif protein 16	2.38
PSMC2	5701	Proteasome (prosome, macropain) 26S subunit, ATPase, 2	2.17
POLH	5429	Polymerase (DNA directed), eta	2.40

a Fold change values refer to the expression in non-irradiated control DCs.

**Table 2:** Downregulated genes in UV irradiated DCs vs non irradiated DCs.

Gene symbol	Access GeneID	Name	aFold change (log 2)
TFEC	22797	Transcription factor EC	−2.11
PPFIBP2	8495	PTPRF interacting protein, protein binding 2	−2.37
LUC7L2	51631	LUC7-like 2 (S. cerevisiae)	−2.58
IFIT1	3434	Interferon-induced protein with tetratricopeptide repeats 1	−2.01
WDR67	93594	WD repeat domain 67	−2.59
INPP5A	3632	Inositol polyphosphate-5-phosphatase, 40kDa	−2.04
TNS	7145	Tensin 1	−2.08
ICOSL	23308	Inducible T-cell co-stimulator ligand	−2.63
MYLK3	91807	Myosin light chain kinase 3	−2.66
CTBP2	1488	C-terminal binding protein 2	−2.17
SFRS3	6428	Splicing factor, arginine/serine-rich 3	−2.16
TTC39B	158219	Tetratricopeptide repeat domain 39B	−2.44
RNGTT	8732	RNA guanylyltransferase and 5′-phosphatase	−2.24
FNDC3B	64778	Fibronectin type III domain containing 3B	−2.31
HNRPLL	92906	Heterogeneous nuclear ribonucleoprotein L-like	−2.39
MITF	4286	Microphtalmia-associated transcription factor	−2.26
TBC1D9	23158	TBC1 domain family, member 9 (with GRAM domain)	−2.26
WWOX	51741	WW domain containing oxidoreductase	−2.86
CAB39	51719	Calcium binding protein 39	−2.46
RASGRP1	10125	RAS guanyl releasing protein 1	−2.50
TAOK3	51347	TAO kinase 3	−2.54
UBE3A	7337	Ubiquitin protein ligase E3A	−3.31
TRIM9	114088	Tripartite motif-containing 9	−3.03
HIVEP2	3097	Human immunodeficiency virus type I enhancer binding protein	−4.35

a Fold change values refer to the expression in non-irradiated control DCs

As expected, several genes typical of UV-irradiation damage were up-regulated, for example, genes involved in DNA damage and p53 signalling, including GDF15, FDXR, POLH, PCNA, and PMAIP1 (Table 1), in accord with published results for human lymphoblastoid cells and human melanocytes [15], [16] confirming the robustness of this system.

Using Gene Ontology designations (WebGestalt), it was observed that in several functional groups, including DNA metabolism, Cell Cycle, Response to stress and Immune Process, a significant percentage of differentially expressed genes was up-regulated with p value<0.05. Other functional groups, including RNA metabolism and genes involved in Cell Motility mainly had down-regulated genes (Tables 1 and 2, and data not shown).

**Figure 1:**
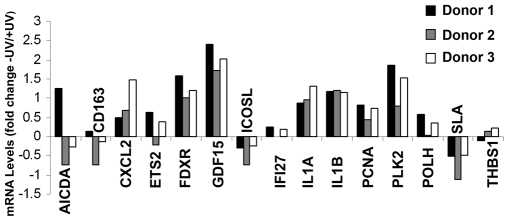
Analysis of gene expression in UV-irradiated human DCs by RT-PCR. Real-time semiquantitative TaqMan RT-PCR was performed to validate differential gene expression induced by UV irradiation in human monocyte-derived dendritic cells (DCs) in culture. DCs (obtained from 3 donors) were untreated or exposed to solar-simulated UV radiation (3.7 J/cm^2^ UVA+0.3 J/cm^2^ UVB). Total RNA was extracted after a further 6 h in culture. Primer sequences are shown in Table S1. Expression levels were normalized to *18s* RNA. Bars correspond to log10 of fold down-regulation or up-regulation compared with non-irradiated cells.

To corroborate the microarray results, 15 of the modulated genes were selected for analysis by TaqMan-based RT-PCR. Table S1 lists the primers and probes for the genes selected for this analysis. Among these genes are several reported to play roles in the regulation of the immune response, including CD163, inducible costimulator ligand (ICOSL), IL1, Src-like adapter protein (SLA) and thrombospondin-1 (THBS1). As controls, we included several stress-response genes known to be modulated in response to UV irradiation (GDF15, FDXR, PLK2, PCNA). The RT-PCR analysis was carried out with the same RNA samples used for the microarrays. The results confirm the altered expression of all the genes tested except for AICDA and CD163 (Fig. 1).

### Other gene expression changes

In addition to Webgestalt program, pathway analysis was also performed using Gene Set Enrichment Analysis (GSEA). GSEA is a method that evaluates microarray data at the level of gene sets. Gene sets examined by GSEA include canonical metabolic and signaling pathways and groups of genes previously identified and validated to be up- or down-regulated when cells are given a particular stimulus. The goal of GSEA is to determine whether members of a gene set *A* tend to occur toward the top (or bottom) of the list of genes analysed by microarrays, and ordered by the statistics (Z score) used in the differential expression analysis (list *B*), in which case the gene set is correlated with the phenotypic class distinction. The results of the GSEA analysis allow us to detect the regulation of gene clusters, including genes not detected in our first statistical analysis because of the stringency of the selection procedure. Table S2 shows some of the gene sets that appear enriched in our system (FDR q value<0.05). The data demonstrate that UVA+UVB-irradiated human DCs are enriched for several gene sets regulated (up- or down-) by UV irradiation in keratinocytes and fibroblasts (Table S2). As expected, the UV-irradiated DCs were also enriched for gene sets involved in oxidative stress, inflammation, and p53 signalling. Furthermore, the GSEA analysis showed that several genes up-regulated in UVA/UVB irradiated DCs are also up-regulated in DCs stimulated for 8 h with LPS (Table S2A). The genes down-regulated by UV radiation in DCs included several involved in chemotaxis, integrin signalling, and vesicular transport (Table S2B).

### Differential gene expression in human DCs, compared with human keratinocytes and human melanocytes

**Figure 2:**
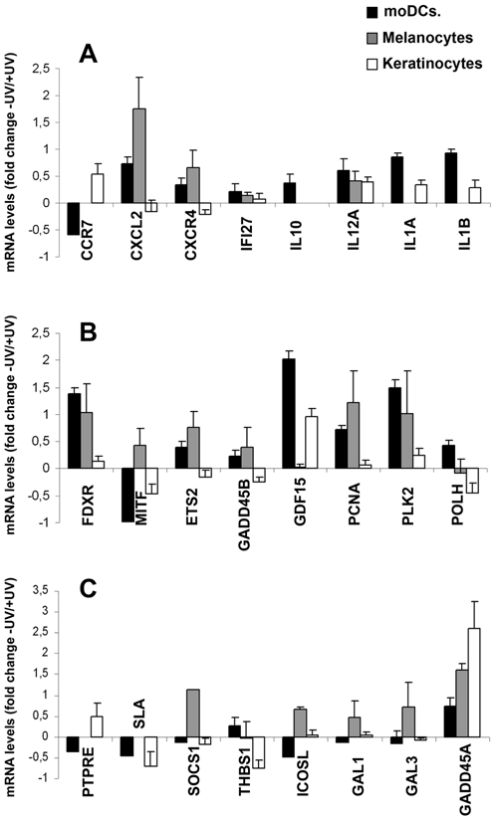
Gene expression induced by UV irradiation in human primary DCs, MCs and KCs. Human primary monocyte-derived DCs (moDCs), melanocytes (MCs) and keratinocytes (KCs) were exposed to solar-simulated UV radiation (3.7 J/cm^2^ UVA+0.3 J/cm^2^ UVB) as in Fig. 1 and total RNA was extracted after a further 6 h in culture. Primer sequences are shown in Table S1. Expression levels were normalized to *18s* RNA. A. Genes encoding cytokines and chemokine receptors. B. Genes related to DNA damage and p53 response. C. Genes potentially involved in immunomodulation. Results are shown as log10 of fold up-regulation or down-regulation in UV-irradiated cells compared with expression in non-irradiated controls. Data correspond to arithmetic mean±SEM.

To identify genes regulated by UV irradiation specifically in DCs, we added a further 11 genes to the set for testing by TaqMan-based RT-PCR: IL-10, GADD45A, GADD45B, Galectin 1, Galectin 3, SLA, CXCR4, SOCS1, PTPRE, CCR7, and IL12A (Table S1). Although the modulation of these genes was not detected by the microarrays experiments, some of them were highlighted by GSEA, others genes like PTPRE, SLA and GADD45A and B were modulated by UV irradiation although with a p value>0.05. All of these genes were chosen mainly because of their potential immunomodulatory roles. The regulation by UV radiation of the complete set of 24 genes was studied in DCs obtained from 6 new donors and compared with human primary keratinocytes and melanocytes. The irradiation dose was the same for the 3 human cells types; cell apoptosis was not detected at time of RT-PCR analysis (data not shown). The 24 genes were classified into three functional groups: i) genes encoding cytokines and chemokine receptors (Fig. 2A); ii) genes related to DNA damage and p53 responses (Fig. 2B); and iii) genes potentially involved in immunomodulation (Fig. 2C). We included the p53 target gene GADD45a with the immunoregulatory genes because there is increasing evidence that it has important functions in the immune system in addition to its roles in cell-cycle arrest, DNA repair, and cell survival [17].

In response to the solar-simulated UV irradiation, human DCs and keratinocytes both up-regulated the expression of genes involved in inflammatory responses (IL-12A, IL-1A and IL-1B) (Fig. 2A). However, whereas DCs down-regulated CCR7 and up-regulated CXCR4 (concurring with our previous results: [14]), the opposite result was observed in human keratinocytes (Fig. 2A). CCR7 gene expression was not detected in melanocytes, although CXCR4 and CXCL2 were induced in these cells. IL-10 expression was induced in human DCs, but was not detected in keratinocytes or melanocytes.

UV radiation produces a variety of lesions in DNA and other cellular targets, triggering complex patterns of stress responses [18]. As expected, several genes involved in these processes were up-regulated in the three cell types, though to different extents; for example FDXR, PCNA, PLK2 and GADD45A (Fig. 2B and 2C). Regulation of other genes was specific to individual cell types: POLH was up-regulated in human DCs but not in identically-treated keratinocytes or melanocytes; and MITF was down-regulated in DC and keratinocytes, but up-regulated in melanocytes.

The greatest concentration of cell-type-specific gene regulation was observed among the immunomodulatory genes (Fig. 2C). The gene encoding protein tyrosine phosphatase epsilon (PTPRE) was down-regulated in UVA+UVB-irradiated DCs, whereas it was up-regulated in identically-treated keratinocytes. Similarly, SLA was down-regulated in human DCs but was not detected in melanocytes under our experimental conditions. Moreover, although SLA was down-regulated in human keratinocytes, it should be noted that its expression in these cells is much lower than in human DCs (data not shown).

Other genes showing a clear differential pattern of expression in DCs compared with melanocytes or keratinocytes include suppressor of cytokine signalling 1 (SOCS1), a member of the STAT-induced STAT inhibitor family [19]. SOCS1 was down-regulated in UV-exposed human DCs and keratinocytes, but was markedly up-regulated in melanocytes (Fig. 2C). Thrombospondin 1 (THBS1) is an antiangiogenic factor with important immunomodulatory properties [20]. In agreement with previous reports, human keratinocytes exposed to UVA+UVB radiation down-regulated THBS1 expression [21]. In contrast, THBS1 mRNA expression in UV-irradiated DCs was increased. THBS1 expression on UV-irradiated melanocytes was very variable and showed no clear pattern of regulation. Inducible costimulator ligand (ICOSL) was down-regulated in human DCs, but up-regulated in melanocytes (Fig. 2C). A similar trend was seen with galectins 1 and 3. Galectins have recently emerged as novel regulators of the inflammatory response and immune cell homeostasis, and are generally thought to act as negative regulators of the immune response [22]. UVA+UVB irradiation resulted in down-regulation of Gal-1 and Gal-3 in human DCs while both transcripts were markedly up-regulated in melanocytes (Fig. 2C). Finally, solar simulated UV irradiation induced a clear increase in the expression of the gene encoding GADD45a in all three cell types (Fig. 2C).

### Protein expression of genes differentially expressed in DCs in response to UV irradiation

**Figure 3:**
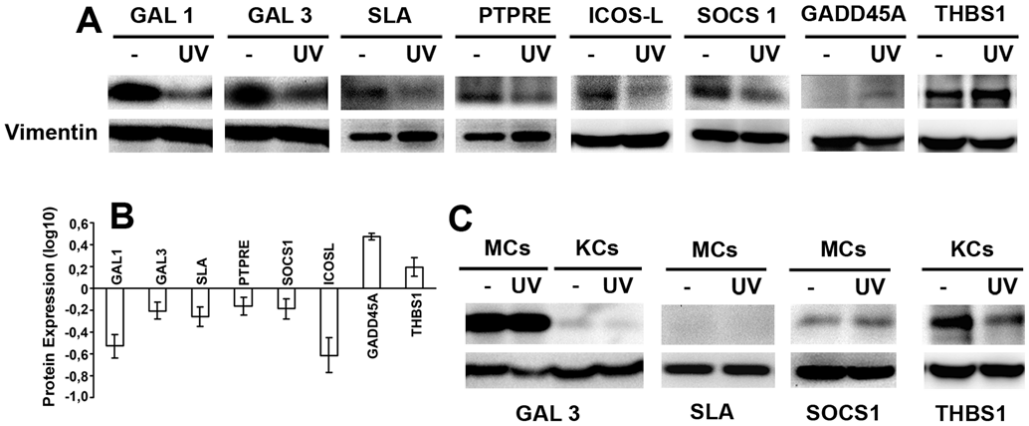
Western blot of selected proteins whose genes are differentially expressed in DCs. A, B. Human primary DCs were exposed to solar-simulated UV radiation as in Fig. 1, and cultured for a further 24 h before lysis and immunoblotting. Experiments were repeated at least three times. Specific protein bands were quantified by densitometry with respect to vimentin (loading control). A. Representative blots. B Results of densitometric analysis, presented as the ratio of expression in irradiated cells to that in non-irradiated cells. Data are the arithmetic means±SEM of three experiments. C. Human MCs and KCs were exposed to solar-simulated UV radiation and analyzed by western blot as in A. Data represents one of three experiments performed.

The functional outcome of altered gene expression results from changes in protein expression. However, protein expression cannot be reliably predicted from changes in mRNA levels. Moreover, the genes found to be differentially expressed have not been studied in detail in DCs. We therefore corroborated the RT-PCR results at protein level by western blot of DC lysates. A representative western blot of proteins encoded by genes regulated in DCs in response to UVA+UVB radiation is shown in Fig. 3A, and quantification of the changed expression relative to untreated cells is shown in Fig. 3B. Consistent with the RT-PCR data, solar-simulated UV-irradiation of human DCs resulted in clear down-regulation of galectin-1, galectin-3, SLA, PTPRE, ICOSL, and SOCS1. Again consistently, UV irradiation induced increases in the amounts of GADD45a and THBS1 proteins. As controls, expression of several proteins was determined in human melanocytes and keratinocytes (Fig. 3C). These blots confirm the absence of SLA protein from melanocytes, as well as the previously reported high expression of galectin-3 in these cells. The previously reported down-regulation of THBS1 in UV-irradiated human keratinocytes was also observed.

**Figure 4:**
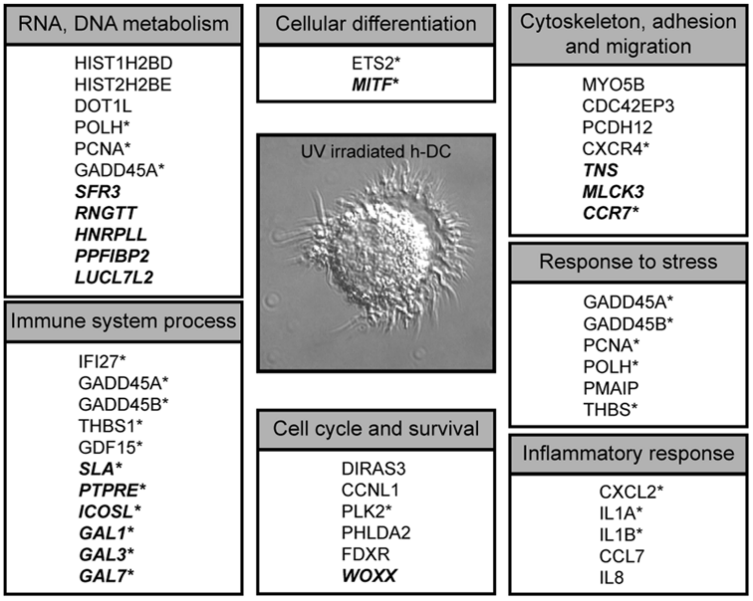
Summary of Genes modified on DCs by Solar-Simulated irradiation. Genes down-regulated or up-regulated in UV irradiated human DCs, compared to non-irradiated DCs were grouped by functional groups, using Gene Ontology designations (WebGestalt) and published literature. Genes bold and italic correspond to down-regulated-genes. (*) Genes selected to corroborate by PCR.

The classification of genes according of their cellular function, following an extensive review of the literature, facilitates the understanding of cellular processes affected in DCs by UV radiation. Fig. 4 shows a summary of the genes modulated by UV solar simulated irradiation clustered by functional groups. In all functional groups showed, the major percentage of genes was up-regulated. However, other functional groups including DNA and RNA Metabolism and Immune System Process mainly had down-regulated genes.

## Discussion

The skin immune system is a highly reactive immunological compartment that is critically involved in the majority of chronic inflammatory skin disorders, including psoriasis and dermatitis [23]. The use of phototherapy to treat inflammatory skin diseases like psoriasis is an example of the immunosuppressive effect of UV radiation [24], [25]. Langerhans cells are well-studied cellular components of the skin immune system. However, the complexity of dermal DCs, more recently identified [26], [27], which are also present in significant numbers in healthy and diseased skin, is only now beginning to be understood [28]. Thus, our objective was to identify genes regulated in monocyte-derived DCs (dermal DCs-like) in response to solar-simulated UV irradiation, focusing our attention on genes involved in the regulation of the immune response. Using Gene Ontology designations and the review of recent published literature, we observed that in addition to the expected genes modulated by UV irradiation, solar simulated irradiation induces the modulation of an important group of genes involved in immune response (Fig. 4). This study has highlighted the regulation of several molecules on DCs that could be participating at the function of these cells.

Transcriptional responses to UV radiation can vary with dose and the time elapsed after irradiation. Also, as has been quoted, besides its immunosuppressive effect, UV radiation is an important stimulus of skin inflammation. The role of UV light on immune response has been studied in different conditions, i.e. chronic or acute UV exposure, UVB only, UVA only, or less frequently with a combination of both, among other parameters. It would be expected that with a particular dose of irradiation, both, induction or repression of the immune response could be present. In this study, we determined the gene modulation at a single time point and a single radiation dose, using a combination of UVA+UVB light. Several genes previously reported to be regulated by UV radiation in other cell types were up-regulated in all three cell types examined for example FDXR, GADD45, GD15, PCNA, PLK2 and IL-1, enabling us to validate our experimental conditions. Solar-simulated radiation of DCs increased the expression of several inflammatory cytokines. Keratinocytes, in addition to DCs, are known to produce and secrete a large number of proinflammatory soluble factors, such as IL-1, IL-5, IL-8, TNF-alpha and prostaglandin E2, thus participating in the onset of inflammation and the induction of chemotaxis to the skin [29], [30]. Accordingly, genes such as IL-1A and IL-B were up-regulated in keratinocytes, whereas mRNA expression of these cytokines was undetectable in melanocytes under our experimental conditions. Besides, the expected functional groups such as response to stress, inflammation and cell cycle and survival, other functional groups relevant for the function of DCs, were detected (Adhesion and Migration, Immune Response Process, and Cell Differentiation, Fig. 4).

The migration altered of LCs and DCs following irradiation has been reported, although the molecules implicated are not completely defined. Previous reports have shown that solar-simulated ultraviolet radiation induces defective chemotaxis by human dendritic cells, associated with increased expression of CXCR4 and a failure to induce CCR7 [14], [31]. Our present results corroborate the altered expression of these genes at the mRNA level in UV-irradiated human DCs, while showing the opposite change in keratinocytes (up-regulation of CCR7 and down-regulation of CXCR4). In addition to the modulated expression of these chemokine receptors, defective chemotaxis by irradiated DCs might be associated with the up-regulation of myosin VB, CDC42 effector protein, and Protocadherin-12 and/or down-regulation of TNS. These molecules could affect not only the motility properties of DCs, but their function. Recently, it has been reported that the disruption of E-cadherin-mediated adhesion induces the maturation of DCs, and generates T cells with a regulatory phenotype [32].

The mechanisms underlying ultraviolet light-induced immunosuppression involve the action of UV-induced regulatory T cells [13] and immunosuppressive mediators such as prostaglandin E_2_, IL-4, and IL-10. In human skin, IL-10 is mainly produced by infiltrating CD11+ macrophages after UV exposure. Recently, it has been described that IL-10 secretion is diminished in UV-irradiated DCs [11], however irradiated DCs produce large amounts of IL-10 after incubation with LPS [14]. Here, we found that IL-10 mRNA expression was up-regulated in UV-irradiated human DCs. On the other hand, we did not detect IL-10 mRNA expression in melanocytes or keratinocytes. In murine skin, IL-10 is predominantly secreted by keratinocytes after UV exposure, whereas the production of this cytokine by human keratinocytes is debated [33]–[35].

Thrombospondin-1 (THBS-1) is a matricellular glycoprotein with anti-angiogenic properties. Accumulating evidence about the role of THBS-1 in immune response has emerged in the last years. In DCs, THBS-1 is known to be an autocrine-negative regulator; human monocyte-derived immature DCs spontaneously produce THBS, which is enhanced by microbial stimuli [36]. The different regulation of THBS-1 by DCs and KCs (induction and inhibition, respectively) after UV exposure could be associated to different roles of THBS-1 in these cells. Keratinocytes from psoriatic skin characterized by an excessive dermal angiogenesis, exhibit a seven-fold reduction in thrombospondin-1 production [37].

Other two genes differentially expressed on DCs after UV irradiation were SLA and PTPRE. SLA is a regulator of TCR levels on thymocytes and regulates B cell development [38], [39]. Our knowledge about these molecules in DCs is limited. Using oligonucleotide microarrays and proteomics, it has been reported that SLA is expressed during DCs differentiation [40]. Our data show that an inflammatory or tolerogenic stimulus is able to modify the expression of SLA on human DCs. Expression of the cytosolic PTPRE form is mainly restricted to hematopoietic tissues, and is up-regulated during differentiation and/or activation of macrophages [41], and its overexpression suppress IL-6 and IL-10-induced JAK-STAT signalling [42]. Importantly, IL-10 production in response to LPS is enhanced in bone marrow-derived macrophages deficient for PTPRE. It would be interesting to assess, whether the PTPRE regulation on DC is associated with the defects of IL-10 production induced by UV light.

Our microarray data show modulation of immune regulatory genes such as ICOSL, SLA, PTPRE or THBS1. Additionally, GSEA identified several other genes (SOCS1, galectin-1 and galectin-3) also involved in the regulation of the immune response. This study has thus allowed us to identify modulation of immunoregulatory molecules that have received very little attention in relation to human DCs, even in terms of protein expression. The identification of proteins selectively regulated in DCs by UV irradiation promises to increase understanding of the biological function of DCs.

Psoriasis is a chronic inflammatory skin disorder whose manifestations are orchestrated by proinflammatory CD4+ T cells that produce either Th1 or Th17 cytokines [43], [44]. A large body of evidence has established a key role for DCs in the initiation and maintenance of psoriasis [45], [46]. Ultraviolet light is an effective treatment for psoriasis. The effectiveness of phototherapy may largely depend on the effects of UV light on the cell cycle and cytokine expression and secretion; however, the cellular targets and effector mechanisms of phototherapy have not been fully elucidated. Further studies will be needed to determine the role of the immunoregulatory molecules identified here in the tolerogenic or inflammatory properties of human DCs. Study of these molecules in a Th1- or Th17-mediated autoimmune disease could help to improve our knowledge of the immunomodulatory properties of DCs, as well as potentially identifying new therapeutic targets.

## Materials and Methods

### Monocyte-derived DCs, keratinocytes and melanocytes

Studies were performed according to the principles of the Declaration of Helsinki and were approved by the local Ethics Committee from Hospital de la Princesa.

Human peripheral blood mononuclear cells (PBMC) were isolated from buffy coats obtained from healthy donors by separation on a Lymphoprep gradient (Nycomed, Oslo, Norway) according to standard procedures. Monocytes were purified from PBMC by a 30 min adherence step at 37°C in RPMI supplemented with 10% fetal calf serum. Nonadherent cells were washed off and the adhered monocytes were immediately subjected to the DC differentiation protocol, as described. Briefly, monocytes were cultured in RPMI, 10% FCS containing IL-4 (10 ng/ml, R&D Systems Inc, Minneapolis, MN USA) and GM-CSF (200 ng/ml, Schering-Plough, Madrid, Spain). Cells were cultured for 6 days, with cytokine re-addition every second day, to obtain a population of immature DC. Phenotypic characteristics of these cells were assessed by flow cytometry on day 6 (HLA-DR^+^, CD1a^+^, CD209^+^, CD14^−^).

Human keratinocytes were obtained from normal skin (foreskin, or skin from abdomen or scalp) as described [47]. Keratinocytes from four donors were kindly provided by JL, Jorcano (CIEMAT Centro de Investigaciones Energéticas, Medioambientales y Tecnológicas, Madrid, Spain) [48]. Briefly, thin sheets of skin were incubated overnight at 4°C in dispase (Roche) in PBS, to enable separation of epidermis and dermis. To obtain single cells, the epidermis was treated with trypsin for 20 min. The epidermal cells were cultured in keratinocyte growth medium (Defined keratinocyte- SFM, Gibco). The medium was replaced every 2 days. Human melanocytes were obtained from foreskin epidermis and cultured in Medium 254CF for melanocytes (Cascade Biologics, Portland, OR USA) as described [49]. Human keratinocyte and melanocyte cultures were split a maximum of four times, and experiments were carried out with cultures at 60–80% confluence.

### Solar-simulated UV radiation

A 1000 watt xenon arc solar simulator (Oriel, USA) equipped with an Oriel 81017 filter (“Colipa”) was used. UVB and UVA irradiance measurements were performed daily using an IL-1700 radiometer (International Light, USA) equipped with SED240/UVB-1/TD and SED033/UVA/TD photodetectors. The radiometer was calibrated with a Solar-Scope spectroradiometer (Solatell, UK).

Human DCs, keratinocytes or melanocytes (3×10^6^ in 0.75 ml Hanḱs balanced salt solution) were irradiated with 3.7 J/cm^2^ UVA+0.3 J/cm^2^ UVB. Immediately after irradiation DCs were cultured for 6 h in RPMI, 10% FCS containing IL-4 (10 ng/ml) and GM-CSF (500 U/ml), and subsequently processed for RNA extraction. Total RNA was extracted with RNeasy Mini Kits (Qiagen).

### Microarray hibridization and signal detection

Total RNA from each sample was labelled, processed and independently hybridized onto a CodeLink human whole genome bioarray (Amersham Biosciences, Uppsala, Sweden) containing 55,000 human gene targets. Hybridizations were made according to the manufacturer's instructions. Slides were scanned with a GenePix Array Scanner and the image was processed using the CodeLink expression analysis software.

### Microarray statistical analysis

Raw intensity values were normalized by the quantile method implemented in the Bioconductor package limma (http://www.bioconductor.org). Data for each experimental set (non-irradiated DCs and UV-irradiated DCs) were filtered according to the following steps: 1) Only spots with two or three quality spots assigned as G (good), or L (low) were selected for subsequent analysis; 2) For genes with only two suitable spots, the third value was assigned as the mean of these two; 3) Values below 0 were assigned a value of 10. After completion of the indicated filtering procedures, data were assigned to the experimental groups: non irradiated DC vs UV-irradiated DC. Statistical analysis for each of the selected groups was carried out by the local pooled error method (package LPE from Bioconductor (http://www.bioconductor.org). P-values obtained were adjusted for multiple hypotheses testing using the step-down false-discovery rate Benjamini-Hochberg procedure implemented in the Bioconductor package multitest. Genes with adjusted P-values below 0.05 were considered to be differentially expressed. All microarray data reported in the manuscript is described in accordance with MIAME guidelines (Data S1).

### Semi-quantitative RT-PCR

Selected DNA microarray results were verified by semi-quantitative TaqMan-based real-time PCR (Universal Human Probe Roche library). Oligonucleotide sequences listed in Table S1 were designed with Roche software for real time PCR and were purchased from OPERON Biotechnologies (Cologne, Germany). Expression levels were normalized to *18s* RNA (cat. Hs99999901_s1, from Applied Biosystems).

### Western blot

After UV irradiation, cells were cultured for a further 24 h after irradiation and then lysed in NP40 1%, desoxicolate 0.5% and SDS 0.1%. Protein extracts were loaded onto 12% (Gal-1, Gal-3, SLA, PTPRE, ICOSL, SOCS1 and GADD45a) or 8% (THBS-1) SDS-PAGE gels. After electroblotting, nitrocellulose membranes were incubated with specific primary antibodies and the corresponding secondary antibodies.

### Functional analysis

Genes were clustered on functional groups using WEB-based GEne SeT AnaLysis Toolkit (WebGestalt) http://bioinfo.vanderbilt.edu/webgestalt/. Gene Set Enrichment Analysis (GSEA) is a computational method that determines whether a defined set of genes shows statistically significant differences in expression between two biological states [50], [51].

## Supporting Information

Table S1 Sequences and probes numbers used for semiquantitative RT-PCR.
(0.05 MB DOC)

Table S2 Gene Sets enriched during UVA+UVB irradiated human DCs according to GSEA. A. Gene Sets (Up-regulated). B. Gene Sets (Down-regulated).
(0.04 MB DOC)

Data S1 Microarray data
(10.44 MB ZIP)
