# Supplementary material for: Identification of Genes Responsive to Solar Simulated UV Radiation in Human Monocyte-Derived Dendritic Cells
Source: PLoS One. 2009 Aug 26;4(8):e6735. doi: 10.1371/journal.pone.0006735 (PMC2727914; doi:10.1371/journal.pone.0006735)
Supplement: Table S1 — Sequences and probes numbers used for semiquantitative RT-PCR. (0.05 MB DOC) [file pone.0006735.s001.doc]

| Gene | Primers | Probe |
| --- | --- | --- |
| IL10 | TGGGGGAGAACCTGAAGAC CCTTGCTCTTGTTTTCACAGG | 30 |
| GADD45A | TTGCAATATGACTTTGGAGGAA CATCCCCCACCTTATCCAT | 19 |
| GADD45B | CATTGTCTCCTGGTCACGA TAGGGGACCCACTGGTTGT | 10 |
| LSGAL1 | AGCGGGAGGCTGTCTTTC CCTGGTCGAAGGTGATGC | 64 |
| LGALS3 | GAGCCTACCCTGCCACTG AGGCAAAGGCAGGTTATAAGG | 3 |
| POLH | TGCATTTACTGTCATCAAGAACTGTGCACAGAGGAAAAGCATTGTG | 63 |
| ICOSLG | GACCGATGCCTCCAACAC GAGCTCCGGTCAAACGTG | 26 |
| ETS2 | CCCCTGTGGCTAACAGTTACA GGACCCATCAAAGGTGTCAA | 20 |
| CCL7 | GAAAGCCTCTGCAGCACTTC AATCTGTAGCAGCAGGTAGTTGAA | 84 |
| IL1A | GGTTGAGTTTAAGCCAATCCA TGCTGACCTAGGCTTGATGA | 6 |
| CD163 | CTGGCGTGACATGTTCTGAT GGCTGCCTCCACCTCTAAGT | 50 |
| IFI27 | CTCAGGAACTCTCCTTCTTTGG TCCGTGGCCTAGAGAGTAAGA | 41 |
| PLK2 | AGATCTCGCGGATTATCGTC TGTCAAATCTGTCATCTCGTAACA | 48 |
| FDXR | GTGACACAGCCGTGATTCTG CTTCGTGATGTCCGTTCTCTC | 21 |
| IL1B | CTGTCCTGCGTGTTGAAAGA TTGGGTAATTTTTGGGATCTACA | 78 |
| GDF15 | CCGGATACTCACGCCAGA AGAGATACGCAGGTGCAGGT | 28 |
| THBS1 | Gccacagttcctgatggag ccatggagaccagccatc | 56 |
| CXCL2 | Catcgaaaagatgctgaaaaatg ttcaggaacagccaccaata | 69 |
| CXCR4 | Ctgtgagcagagggtccag atgaatgtccacctcgcttt | 55 |
| PCNA | Tggagaacttggaaatggaaa gaactggttcattcatctctatgg | 69 |
| SOCS1 | Gccccttctgtaggatggta ctgctgtggagactgcattg | 87 |
| PTPRE | Ggctcacctggttcagga catcaccctttgctgctctt | 6 |
| MITF | Agggagctcacagagtctgaa tgttaaatcttcttcttcgttcaatc | 34 |
| CCR7 | Ggggaaaccaatgaaaagc acctcatcttgacacaggcata | 77 |
| SLA | Tgtcaccttgcgtcagaaga ggggtcctcctgcagtct | 25 |
| IL12A | CACTCCCAAAACCTGCTGAG TCTCTTCAGAAGTGCAAGGGTA | 50 |
