# Supplementary material for: Identification of Genes Responsive to Solar Simulated UV Radiation in Human Monocyte-Derived Dendritic Cells
Source: PLoS One. 2009 Aug 26;4(8):e6735. doi: 10.1371/journal.pone.0006735 (PMC2727914; doi:10.1371/journal.pone.0006735)
Supplement: Table S2 — Gene Sets enriched during UVA+UVB irradiated human DCs according to GSEA. A. Gene Sets (Up-regulated). B. Gene Sets (Down-regulated). (0.04 MB DOC) [file pone.0006735.s002.doc]

A.

| Gene Set Name | Description |
| --- | --- |
| BLEO_HUMAN_LYMP_ HIGH_4HRS_UP | Up-regulated at 4 hours following treatment of human lymphocytes (TK6) with a high dose of bleomycin |
| UVB_NHEK1-C2 | Upregulated by UV-B light in normal human epidermal keratinocytes, |
| HINATA_NFKB_UP | Genes upregulated by NF-kappa B |
| OXSTRESS_BREASTCA_UP | Upregulated by H2O2, Menadione and t-BH in breast cancer cells |
| NAKAJIMA_MCS_UP | Most increased transcripts in activated human and mouse MCs |
| YANG_OSTEOCLAST_SIG | Relative gene expression for osteoclast-associated genes, chemokines, and chemokine receptors |
| LINDSTEDT_DEND_8H VS_48H_UP | Genes up-regulated in DC stimulated for 8 h as compared to DC stimulated for 48 h |
| CYTOKINEPATHWAY | BioCarta |
| TNFA_NFKB_DEP_UP | Up-regulated at any timepoint following TNFa treatment, only with functional NFkB |
| [EGF_HDMEC_UP](http://www.broad.mit.edu/gsea/msigdb/cards/EGF_HDMEC_UP.html) | Up-regulated in human dermal endothelial cells stimulated to proliferate with EGF treatment |
| INFLAMPATHWAY | BioCarta |
| HOHENKIRK_MONOCYTE__DN | Down-regulated mRNAs in monocyte-derived DCs |
| P53GENES_ALL | p53 transcriptional targets |
| IL1RPATHWAY | BioCarta |
| DCPATHWAY | BioCarta |

B.

| Gene Set Name | Description |
| --- | --- |
| UVB_NHEK1_C6 | Downregulated by UV-B light in normal human epidermal keratinocytes |
| SIG_CHEMOTAXIS | Genes related to chemotaxis |
| UVC_TTD-XPCS_COMMON DN | Down-regulated at any timepoint following treatment of both XPB/CS and XPB/TTD fibroblasts with 3 J/m^2 UVC |
| MRNA_PROCESSING | Genes involved in mRNA processing |
| BYSTROM_IL5_DN | Genes downregulated in mouse bone marrow in response to interleukin-5. |
| BRENTANI_TRANSPORT_OF_VESICLES | Cancer related genes involved in vesicle transport |
| ST_INTEGRIN_SIGNALINGPATHWAY | Integrins mediate cell growth, survival, and migration. |
